# Rehabilitation Towards Functional Independence in a Patient With Intertrochanteric Fracture and Paraplegia: A Case Report

**DOI:** 10.7759/cureus.32689

**Published:** 2022-12-19

**Authors:** Palash R Satone, Abhishek Daf, Avanti A Gachake, Neha V Chitale, Pratik Phansopkar

**Affiliations:** 1 Department of Physiotherapy, Ravi Nair Physiotherapy College, Datta Meghe Institute of Medical Sciences, Wardha, IND

**Keywords:** physical therapy, rehabilitation, femur intertrochanteric fracture, paraplegia, spinal cord injury

## Abstract

Traumatic spinal cord injury (SCI) causes significant neurological deficits that adversely affect the quality of life of patients and caregivers. Patients with SCI present with the symptoms of weakness and loss of sensations in the limbs. Motor deficits may occur in the form of paraplegia, hemiplegia, or quadriplegia. Patients remain immobilized for a prolonged period which may lead to complications like muscle wasting, atrophy, joint stiffness, contractures, bed sores, and osteoporosis. Patients are prone to fractures owing to osteoporosis. The fractures may occur even due to trivial trauma. Our case report presents the case of a 45-year-old male patient who is already diagnosed with paraplegia having a history of SCI one-and-a-half years ago. He has recent history of intertrochanteric (IT) fracture that occurs during the transfer from bed to wheelchair. Admitted to the hospital with a complaint of a popping sound along with low back pain. Further investigation and management were started.

## Introduction

Spinal cord injury (SCI) is defined as damage to the spinal cord that temporarily or permanently causes changes in its function [1]. Patients with spinal cord injuries suffer tremendous socio-economic difficulties in life [2]. The primary cause of SCI is road traffic accidents which is about 38% and other cause includes fall or trauma injury about 30%, 9% sports-related injuries, 13% by violence, and 5% by surgical and medical cause [3]. SCI is of two types traumatic and non-traumatic. Clinical syndromes of SCI are Brown Sequard syndrome, central cord syndrome, posterior cord syndrome, anterior cord syndrome, and cauda equina syndrome. A period of areflexia called spinal shock appears for approximately 24 hours after the injury. The reflexes gradually return in the next three days, and finally, hyperreflexia occurs after six months of injury. The loss or impairment of sensory and motor function in the lumbar, thoracic, or sacral portions of the spinal cord (except cervical) occurs due to damage to neurological components in the spinal canal. Autonomic dysreflexia is one of the red flags in SCI patients [4].

Spinal cord injuries accompany many medical complications at every stage. Due to sensory loss, complications such as deep vein thrombosis, ureteric colic, pyelonephritis, and reduction in bone density may be present without showing typical signs and symptoms and are thus mostly overlooked [5]. The reduction in bone density makes them susceptible to fractures even due to low-impact injuries. The fractures may occur spontaneously or during the transfer from bed to a wheelchair and vice-versa [6]. Patients with paraplegia are more prone to lower extremity fractures than those with quadriplegia since paraplegic patients are functionally more active. The majority of fractures occur during seemingly mild activity such as transfer and positioning, and rest is the outcome of a violent accident [7]. 

Intertrochanteric (IT) fracture is an extracapsular fracture that develops at the level of the greater and lesser trochanters. This fracture has multiple classifications; the orthopedic trauma association classification is most preferred among others. This problem commonly occurs in the older population as a result of trivial falls. In the younger population, a violent blow to the hip causes an IT fracture. The patient comes with the complaint of pain in the groin region and inability to move the leg followed by a history of falls or accidents [8]. The management of IT fractures involves internal fixation with a percutaneous compression plate, dynamic hip screw (DHS), sliding plate, compression hip screw, proximal femoral nailing, and a less invasive stabilization system. DHS is the most common method to treat IT fractures as it is a more suitable and safe method. But nowadays proximal femoral nailing is most commonly used than DHS because it has fewer complications and more merits such as less blood loss, reduced duration of surgery, early weight bearing and mobilization, reduce hospital stay and reduce risk of infection. The goal of treatment is to return the patient back to his pre-fracture state by preventing complications and making the patient functionally independent. Early physiotherapy management reduces the risk of prolonged bed rest and increases the chances of early mobility and independent functional activity [9].

## Case presentation

A 45-year-old male farmer with a known case of paraplegia for one-and-a-half years was brought to the hospital with complaints of a popping or cracking sound from the hip and sudden low back pain while transferring the patient from the bed to the wheelchair. The patient went to the hospital for further investigation and management. The X-ray revealed the right side intertrochanteric fracture of the femur. Then, the fracture was managed surgically for the same. The patient also gave a history of SCI after the fall from the tree one year back. He sustained wedge compression thoracic vertebrae (D7) fracture following the fall. He was taken to the nearby hospital immediately where the vertebrae were fixed with an implant. The patient was bedridden for one year. The motor and sensory functions were not present in both the lower extremities. He also had bladder and bowel dysfunctions for one year. He developed bed sores over his buttocks six months before.

Clinical findings

We performed a physical examination on postoperative day three. During observation, the patient was in a supine lying position with his back appropriately supported. On examining the chest, there was a reduction in chest mobility. There was the presence of swelling over the right hip. There was an impairment in the motor function below the D7 level. There was a reduction in the range of motion (ROM) in both lower limbs as shown in Table 1. On neurological examination, muscle strength was grade 0 on manual muscle testing, deep tendon reflexes were absent in both the lower limbs and the muscles in both the lower limbs were flaccid. The strength in the upper limb was 4/5.

**Table 1:** Range of motion of joints

Joints	Right (operated side)		Left	
	Active	Passive	Active	Passive
Hip flexion	0	0-20	0	0-111
Hip extension	0	0	0	0-11
Knee flexion	0	0-20	0	0-130
Knee extension	0	10-0	0	130-0
Hip adduction	0	0	0	0-25
Hip abduction	0	0	0	0-45
Ankle dorsiflexion	0	0-15	0	0-25
Ankle plantarflexion	0	0-20	0	0-40

As a further course of examination, we performed limb length measurement and found a shortening of the right lower limb as shown in Table 2.

**Table 2:** Limb length measurement of both the lower limbs cm: Centimeters

Lower limb side	Length (in centimeters)
Left	85 cm
Right	81 cm

Investigations

**Figure 1:**
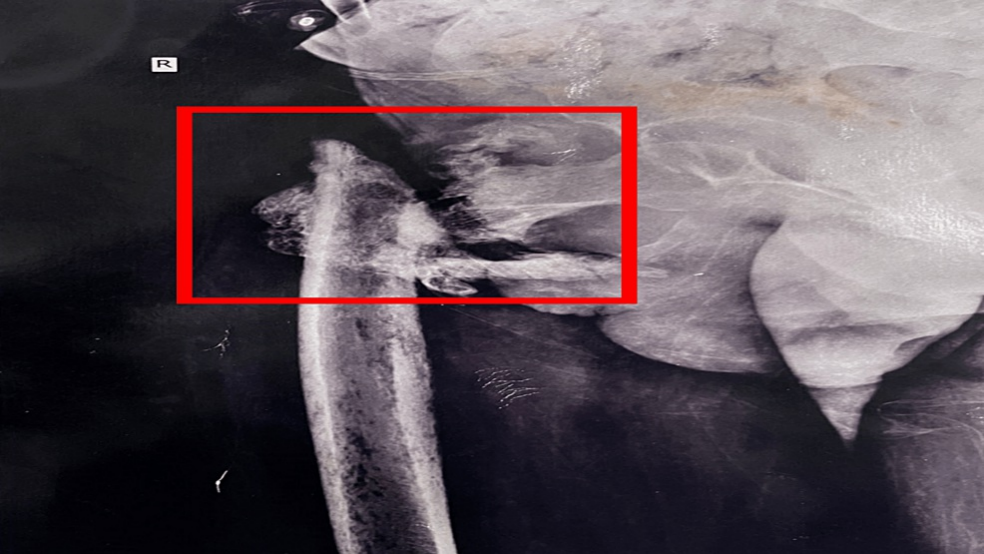
Preoperative X-ray of the anteroposterior view of the right hip

**Figure 2:**
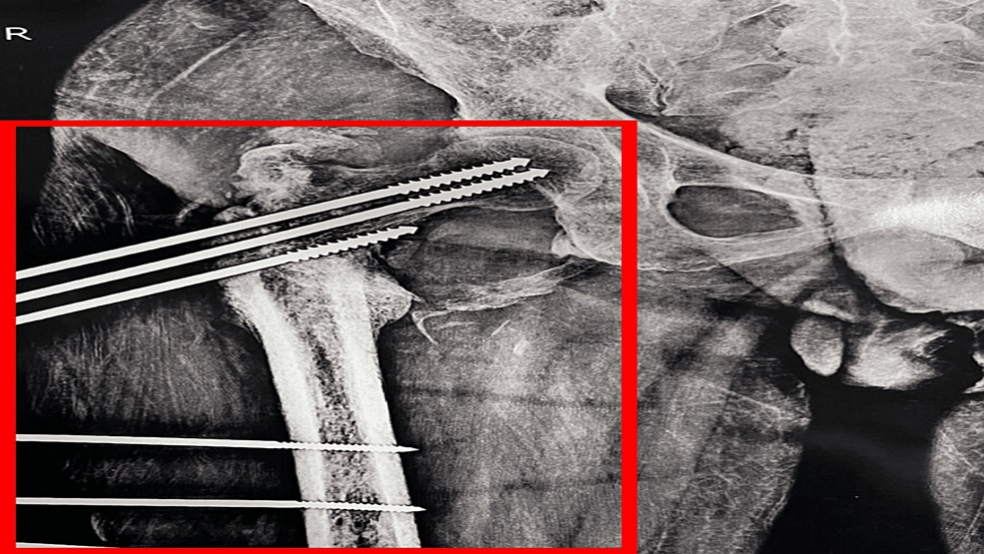
Postoperative X-ray image The image is showing an intertrochanteric fracture fixed with simple external fixation

**Figure 3:**
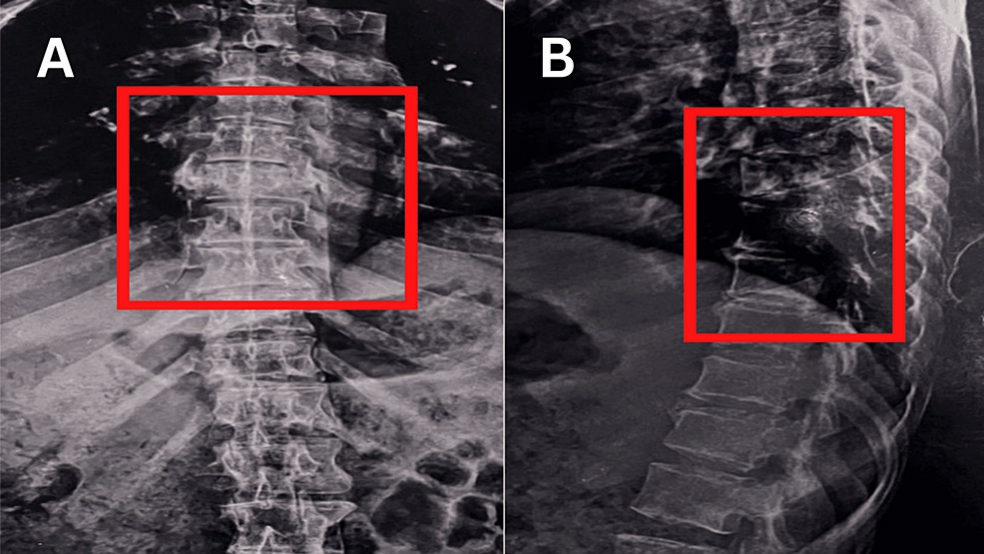
The one-year-old spine X-ray image The image is showing a D7 wedge compression fracture. (A) Postero-anterior view (B) Lateral view D7: Seventh dorsal vertebrae

The preoperative X-ray of the anteroposterior view of the right hip revealed an intertrochanteric fracture as shown in Figure 1. Figure 2 displays a postoperative X-ray showing an intertrochanteric fracture fixed with simple external fixation. Figure 3 displays a one-year-old X-ray of the spine showing a D7 wedge compression fracture.

Physiotherapeutic intervention

The goals of physiotherapy management were patient education, swelling reduction, addressing limb length discrepancy, preventing postoperative pulmonary and cardiovascular complications, maintaining range of motion, improving muscle tone, and maintaining the strength of the upper extremities. Table 3 shows the structured physiotherapy protocol for the given case. Week-wise protocol for the right side fractured lower limb is shown in Table 4.

**Table 3:** The structured physiotherapy protocol for the given case PROM: Passive range of motion

	Goals of rehabilitation	Therapeutic intervention	Rehabilitation rationale
1	Patient Education	Educate the patient about the condition prevention of bedsores and the importance of exercise	Changing the position of the patient every two hours
2	Reduce swelling	Applying a Crepe bandage in the figure of four pattern from knee to hip and elevating the leg at 15^0 ^ with the knee extended, the knee and foot will be supported by a pillow	To reduce swelling by compression and elevation
3	Correction of limb length discrepancy	Suitable Shoe modification with a height of 4 cm in such a way both limb lengths will be the same	To reduce or prevent limb length discrepancy
4	Prevent complications of the pulmonary and cardiovascular system	Initially, an active cycle of breathing technique for 10 repetitions as the condition progresses thoracic expansion exercises and spirometry started (10 repetitions of each two hourly)	To maintain and improve lung compliance
		Upper limb mobility exercises, Cycle ergometer (two times a day)	To prevent cardiovascular complications and improve endurance
5	To maintain the hip and knee joints' Range of motion	PROM exercise for Hip and knee in supine lying to the non-fracture side. 15 days – 20 repetitions x 3 sets. Progression: 30 repetition x 3 sets	flexion-extension of knee, flexion-extension, adduction-abduction and extension of hip (20 repetitions x 3 sets)
		PROM exercise of knee and Hip in supine lying on the affected side	Ankle dorsiflexion 20^o^ Ankle plantarflexion 15^o^
6	To maintain the strength of the upper extremities	(1) Shoulder and elbow flexion-extension, shoulder adduction-abduction with 1kg weight cuff	Increasing the weight as strength progress help with ambulation or further rehab (10 repetitions x 3 sets each)
		(2) Holding the dumbbells for five seconds without support	
7	To activation of muscles	Functional electrical stimulation	Quadriceps, Hamstring, and calf muscles (30 reps x 3 sets)
8	Roods approach	Facilitatory technique	–To improve the tone of muscles from a flaccid state. – fast brushing Icing, stroking, tapping, quick stretch
9	Proprioceptive neuromuscular facilitation	Proprioceptive neuromuscular facilitation: (1) strengthening (2) stretching	Proprioceptive Neuromuscular Facilitation to the lower limb bilaterally which improve mobility stability and strengthen lower limb musculature (10 reps x 3 sets)

**Table 4:** The week-wise protocol for right side fractured lower limb

Week	Rehabilitation (Fracture limb)
Week 0-2	No movement in the hip before two weeks, Positioning the leg by keeping the pillow in between leg to prevent bed sores, passive ankle toe movements with 10 repetitions, three sets
Week 2-3	Small range passive movements in hip and knee to fracture limb are started, assisted straight leg raise upto 15-25 ° with 10 repetitions, knee swinging while sitting on edge of the bed for 5-10 minutes
Week 4-8	Passive Hip flexion up to 90 °
Week 8 - above	Increase in passive range of motion exercise repetitions and intensity, passive weight bearing

Outcome measures and follow-up

We used the ASIA (the American spinal injury association impairment) scale for this case as an outcome measure showing the patient's impairment score with spinal cord injury as shown in Table 5.

**Table 5:** The pre-intervention score on the ASIA scale with neurological level D7 ASIA: American spinal cord injury association impairment scale; D7: Seventh Dorsal Vertebrae

Component	Pre-intervention score
Motor component	30
Sensory component	50

We followed up with the patient after 10 weeks of rehabilitation which shows improvement as shown in Table 6.

**Table 6:** Outcome measures with post-rehabilitation scores ASIA: American spinal cord injury association impairment scale; D7: Seventh Dorsal Vertebrae

Outcome measures	Post Interventions
Swelling on right thigh	No swelling appears after 14 days
Limb length discrepancy	Limb length appears equal after shoe modification
Passive Range of motion	Hip flexion-90 ° , Knee flexion-100 ° , Knee extension-100 ° , Hip abduction-25 °
Modified Ashworth scale	Grade 1- a slight increase in muscle tone manifested by catch and release at the end of the range of motion
Manual muscle testing	Grade 1- flicker of contraction
American spinal injury association scale (ASIA)	Pre-intervention score: Sensory component -50, Motor component-30, Post-intervention score: Sensory component - 72, Motor component-34, and neurological level was D7.

## Discussion

A fragile IT fracture in a patient with SCI occurs due to a reduction in bone mineral density. This fracture occurs during the transfers and positioning. The management of this patient aimed to prevent the complications like bed sores, joint stiffness, muscle wasting and atrophy, contractures, and pulmonary complications [10]. Our study presented the case of a paraplegic patient who sustained an IT fracture while transferring from a bed to a wheelchair. The fracture was managed surgically by an external fixator. We started physiotherapy management from postoperative day three and applied preventive measures to reduce associated complications. For the management of paraplegia, rehabilitation aims to maintain or improve residual strength or prevent the decline in the muscle strength of the upper extremities in the acute phase. A daily strengthening training exercise for the upper extremities helps in upcoming rehabilitation [11].

Shroff et al. [12] concluded that physiotherapy assists people with SCI in functioning with their injuries daily. It entails mobilization exercises as well as muscle and nerve stimulation below the level of the injury. Furthermore, it may aid in the restoration of disused muscle function. Kumar et al. [13] concluded in their study that osteoporosis and paraplegia are frequently linked, and fractures can result from seemingly benign events such as forceful passive ROM of the joints. The surgeon, physiotherapists, and patients' companions should understand this consequence and refrain from using force. Force during the passive range of exercise should be firm, slow, and below the limit that will prevent fractures in spinal cord injury patients during rehabilitation. Early mobilization, embedded patient rehabilitation, and home-based rehabilitation help the patient to strengthen the muscles, reduce pain, and bring back to their activities of daily living (ADL) or make a faster recovery and application of some preventive measures to reduce complications which facilitates the patient recovery and make fit for doing their ADL [14].

## Conclusions

Patients with paraplegia are susceptible to sustaining IT fractures during transfers from bed to wheelchair and vice-versa. In this case report, we have shown the major role of physiotherapy in the postoperative management of fragile fractures in paraplegic patients to prevent complications and improve available muscle strength. The primary goal is to manage the fracture once it heals and then start rehabilitation for paraplegia which includes muscle tone facilitation and strengthening. This has played a significant role in patient independence. Outcomes from this case report can be helpful in further clinical practices.
